# Granular cell tumor of the trachea as a rare cause of dyspnea in a young woman

**DOI:** 10.1016/j.rmcr.2019.100961

**Published:** 2019-10-25

**Authors:** A. Rizzo, E.D. Serban, A.D. Ricci, M. Nannini, M. Saponara, A. Cancellieri, D. Paioli, R. Trisolini, M.A. Pantaleo

**Affiliations:** a Department of Specialized, Experimental and Diagnostic Medicine, Sant’Orsola-Malpighi Hospital, University of Bologna, Via Massarenti 9, 40138, Bologna, Italy; b Pathology Unit, S.Orsola-Malpighi Hospital, Via Massarenti 9, 40138, Bologna, Italy; c Interventional Pulmonology Unit, Policlinico Sant'Orsola-Malpighi and Ospedale Maggiore, Bologna, Italy

**Keywords:** Tracheal granular cell tumor, Tracheal malignancies, Trachea, S-100, Airway tumors

## Abstract

Tracheal granular cell tumors are rare neurogenic neoplasms characterized by an indolent behavior. We report the case of a young woman affected by this tumor with non-specific clinical presentation. We performed a literature search in order to identify all the cases of tracheal granular cell tumor and to summarize the current state of knowledge about this rare disease.

## 1 Introduction

Granular cell tumors (GCT) are rare tumors of neurogenic origin [1]. Although these neoplasms can occur in any part of the body, GCTs are rarely reported in the laryngotracheal region [2]. In tracheal GCTs, treatment can vary from simple observation to tracheal resection or endoscopic excision assisted by laser, electrosurgery or argon plasma coagulation [3]. In this study, we present the case of a woman affected by tracheal GCT who presented with acute dyspnea. We conducted a literature search and we found 42 cases of GCT of the trachea including clinical, epidemiological and therapeutic characteristics, summarizing the current state of knowledge about this rare tumor.

## 2 Case presentation

**Fig. 1:**
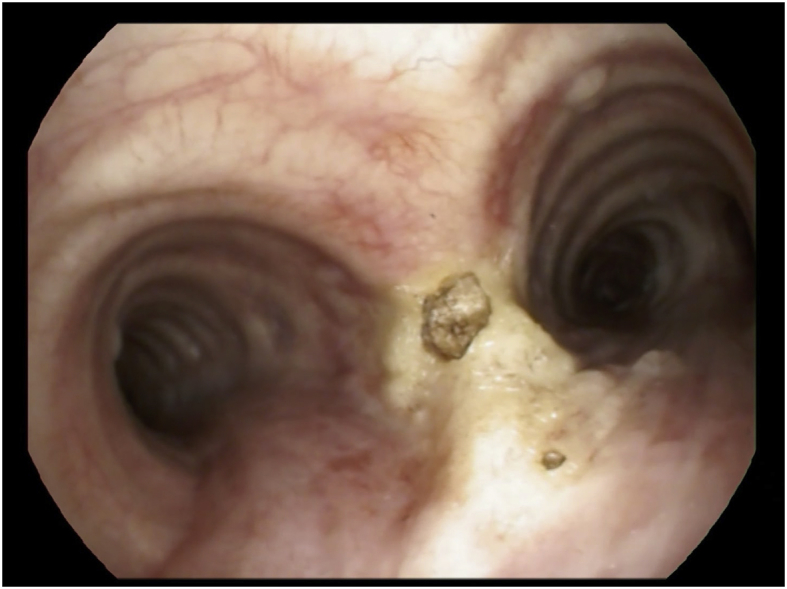

A 42-year-old former smoker woman (30 pack-years) presented to the Emergency Department with acute onset of dyspnea, cough and fever. The patient had no comorbidities and the physical exam was within normal limits. In order to exclude a pulmonary thromboembolism, a computed tomography angiography was performed, revealing bilateral hilar and mediastinal lymphadenopathy and various areas of ground glass of both the inferior lobes. Several blood tests were done in order to exclude an infectious disease; the tests were negative, including QuantiFERON-TB Gold test. Bronchoscopy showed a marked thickening of the tracheal carina extending for a few millimeters to the medial aspect of both mainstem bronchi (Fig. 1).

**Fig. 2:**
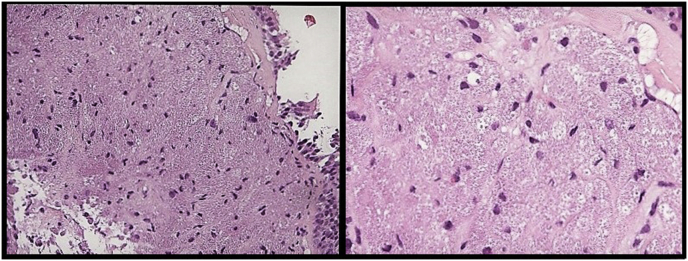
H&E staining reveal large cells with cytoplasmic granules.

**Fig. 3:**
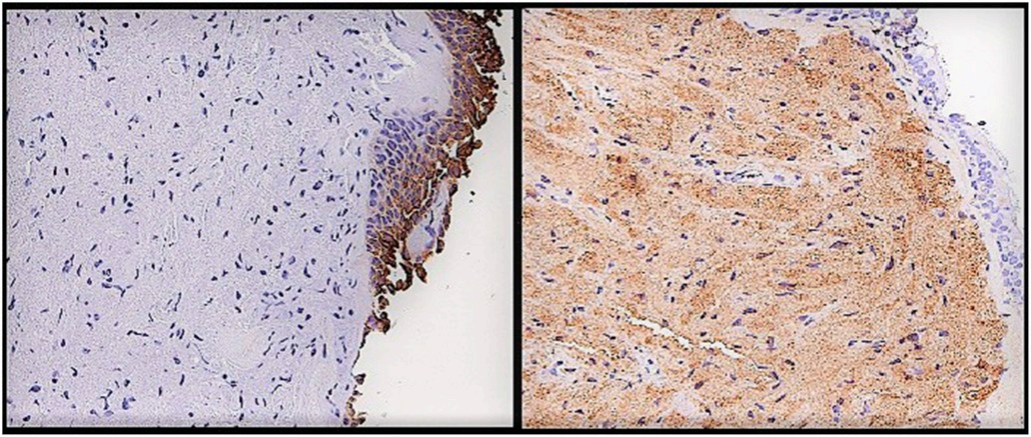
Immunohistochemical staining: tumor cells positive for NSE, S-100 and CD68.

**Fig. 4:**
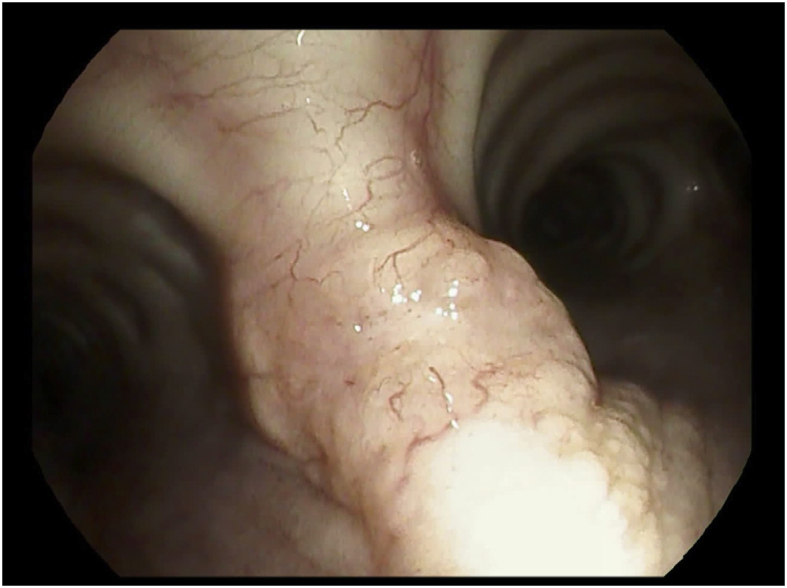

Histopathology examination of the bronchial biopsies performed in this area revealed sheets of epithelioid cells with eosinophilic granular cytoplasm, positive for S100 and NSE and negative for CAM5.2 (Fig. 2; Fig. 3), consistent with a diagnosis of GCT. An endobronchial ultrasound-guided transbronchial needle aspiration (EBUS-TBNA) of the lymph node stations #4R, #7 and #11Rs was performed and the cytopathological examination suggested their “reactive” nature by showing a predominance of lymphocytes. Culture of bronchoalveolar lavage (BAL) for common bacteria, fungi and mycobacteria proved negative. The tumor was ablated with Nd:YAG laser (Fig. 4) during rigid bronchoscopy under general anesthesia and the patient is currently free of disease 12 months after treatment.

## 3 Discussion

GCTs (also known as Abrikossoff tumors) were reported by Weber in 1854 [4] and later described by Abrikossoff in 1926 [5]. They were originally considered muscle tumors and called myoblastomas on the basis of a supposed myogenic origin. Due to their ultrastructural and immunohistochemical characteristics, in the last 30 years GCTs have been identified as neural lesions deriving from Schwann cells [1]. These neoplasms are characterized by a very slow growth rate and a benign behavior, reported in up to the 98% of cases; cases of malignant GCTs have been documented in literature although none of these variants have never been reported in trachea [6]. GCTs usually involve skin, breast and especially the gastrointestinal tract (e.g. oral cavity, tongue and esophagus) although they can occur anywhere in the body [7]. GCTs represent a very rare finding in the respiratory tract, particularly in the laryngotracheal region [8] as evidenced by the only 42 cases, including our patient, of tracheal GCTs reported in literature up to now.

We performed a literature search for the present study. The search included PubMed, EMBASE and Google Scholar, and used the following search terms: “tracheal granular cell tumor”, “granular cell tumor”, “rare airway tumors”, “tracheal malignancies”. The inclusion criteria were as follows: all types of articles, articles published in PubMed and related only to humans. Articles for which full text was not available were excluded. The Medline search of GCT of the trachea found altogether 41 cases. Overall, forty-two cases of GCT have been identified. The clinical and pathological findings of all cases are summarized in Table 1. The age range was 6–64 years, with a mean and a median age of 33 years and 32 years, respectively. Thirty-five of 42 (83,3%) patients were women and 22 of 30 (73,3%) were of African or African American origin and 2 patients were Asians. The mean size of the neoplasm at the time of diagnosis was 2.27 cm, with a range from 0.45 to 6 cm in its large axis. Seven of 42 (16,7%) of the reported cases of GCTs were multiple. As regards the site, 15 of 42 tumors were intrathoracic, 26 cervical. In one case, GTCs were both cervical and intrathoracic in location. Our search listed 31 intraluminal neoplasms and 8 extraluminal. Three of 42 tumors were both intraluminal and extraluminal. Twenty-four of 42 patients received tracheal resection while 7 tumors were endoscopically excised. One patient was treated with external beam radiation. Of the 24 tracheal resections, no recurrences have been reported; the rate of recurrence in endoscopically resected patients was 29%, with 2 of 7 cases. Of the 24 GCTs treated with tracheal resection, 3 patients (12,5%) died for postoperative complications. Five of 24 patients who underwent tracheal resection also received thyroid lobectomy; two cases were treated with electrosurgery and argon plasma coagulation.

**Table 1:** Published data about Tracheal Granular Cell Tumors.

Source	Age	Sex	Race	Size (cm)	Solitary/Multiple	Location	Intra/extraluminal	Therapy	Results/Follow-up
Archer	56	F	Black	2.8	S	Intrathoracic	Intra	Tracheal resection	Died postoperatively
Benson	22	F	NA	0.5	M	Intrathoracic	Intra	Endoscopic excision	Residual disease/8 months
Vance	NA	NA	NA	NA	S	Intrathoracic	Intra	Endoscopic excision	NED/7 months
Sargent	50	F	Black	NA	S	Intrathoracic	Intra	Radiation therapy	DWD/5 months
Benisch [15]	25	F	Black	1.5	S	Cervical	Intra	Tracheal resection	NED/15 months
Krouse	54	F	Black	2.5; 0.5	M	Cervical and intrathoracic	Extra	None	Postmortem finding
Thawley	33	F	Black	NA	S	Cervical	Extra	Thyroid lobectomy, tracheal wall shaving	Residual disease/8 years
Carnalis	45	F	NA	5.5	S	Cervical with posterior wall erosion	Intra and extra	Tracheal resection	NED/3 years
Frable	38	F	Black	NA	S	Cervical	Intra	Endoscopic excision	NED/5 years
O'Connell	36	F	Black	NA	M	Cervical	Intra	Tracheal resection	Died postoperatively
Polack	29	F	Black	2	S	Cervical	Extra	Local excision, tracheal shaving	NED/4 weeks
Subbuswamy	35	F	Black	2.5	S	Intrathoracic	Intra	None	Postmortem finding
Daniel	31	F	NA	1.5	S	Intrathoracic	Intra	Tracheal resection	NED/4 months
Dunaway	6	F	Black	2	M	Cervical	Intra	Endoscopic excision	Residual disease/9 years
Cech	41	M	White	1.5	S	Intrathoracic	Intra	Tracheal resection	NED/6 years
McLain	26	M	Black	2	M	Cervical	Intra	Tracheal resection	NED/9 months
Mikaelian	20	F	Black	2	S	Cervical	Intra	Tracheal resection	NED/2 years
Mikaelian	30	F	Black	3	S	Intrathoracic	Intra	Tracheal resection	NED/5 years
Thaller	31	F	Black	6	S	Cervical	Intra	Endoscopic excision	NED/6 weeks
Muthuswamy	26	F	Black	2	S	Intrathoracic	Intra	Tracheal resection	NED/1 year
Alessi	33	F	NA	NA	S	Cervical	Extra	Partial tracheal resection	NED/7 years
Alessi	37	F	NA	3	S	Cervical	Intra	Tracheal resection, thyroid lobectomy	NED/2 years
Solomons	10	M	NA	NA	S	Cervical	Intra	Partial tracheal resection	Died postoperatively
Burton [9]	14	F	Black	NA	M	Cervical	Intra	Tracheal resection	NED/4 months
Burton [9]	38	F	White	NA	S	Intrathoracic	Intra	Tracheal resection	NED/1 year
Burton [9]	37	F	Black	3	S	Cervical	Extra	Partial tracheal resection, thyroid lobectomy	NED/1 year
Burton [9]	19	F	NA	1.5	S	Cervical	Intra	Endoscopic excision	NED/8 years
Burton [9]	43	F	Black	4	S	Cervical	Intra and extra	Tracheal resection, thyroid lobectomy	NED/6 months
Burton [9]	25	M	Black	1.5	S	Intrathoracic	Intra	Tracheal resection	NED/2 years
Spandow [10]	12	M	NA	NA	S	Cervical	Intra	Tracheal resection	NED/18 months
Thomas	46	F	NA	0.45	M	Cervical	Intra and extra	Tracheal resection	NA
Raymond	50	M	Black	4.8	S	Intrathoracic	Extra	Tracheal resection	NED/20 months
Frenckner	28	F	White	NA	S	Cervical	Intra	Transtracheal enucleation	NED/3 years
Desai [1]	10	F	NA	NA	S	Cervical	Intra	Tracheal excision	NED/10 years
Kintanar [13]	35	F	NA	2.2	S	Cervical	Extra	Partial tracheal resection, thyroid lobectomy	NA
Ipakchi [3]	22	F	Black	3	S	Cervical	Intra	Endoscopic resection	NED/18 months
Lee [8]	45	F	Asian	3	S	Cervical	Extra	Tracheal resection, thyroid lobectomy	NED/13 months
Guarnieri [16]	64	F	White	1.3	S	Cervical	Intra	Electrosurgery and argon plasma coagulation	NA
Bekteshi [11]	37	F	Black	0.6	S	Intrathoracic	Intra	Electrosurgery and argon plasma coagulation	NED/3 months
Joung [20]	20	F	Asian	NA	S	Intrathoracic	Intra	None	NED/2 years
Stieglitz	14	F	White	1.5	S	Cervical	Intra	Tracheal resection	NED/3 years
Current study	42	F	White	0.5	S	Intrathoracic	Intra	Nd: YAG laser ablation	NED/1 year

NA: not available; F: female; M: male; S: solitary; M: multiple; Intra: intraluminal; Extra: extraluminal; NED: no evidence of disease; DWD: died with disease.

As stated before, median age at diagnosis of tracheal GCTs appears to be around 33 years, with a higher incidence in African and African American women (Table 2). Nevertheless, there are some remarkable exceptions to this general rule, as indicated by several cases of tracheal GCTs in pediatric age [9,10]. No specific risk factors are currently linked to the diagnosis of tracheal GCT although in most cases active smoking or a history of smoking is reported [11]. Most GCTs are found in females supporting a hormonal relationship between gender and disease [12]. Because of the small number of patients and the missing data, it is not possible to determine an established and definitive correlation between hormonal factors and tracheal GCTs although the incidence of GCT in hyperestrogenic state is reported by several authors [[13], [14], [15]]. Our search could support this correlation between gender and tracheal GCT since the 85,4% of patients affected are female.

**Table 2:** Summary of chief characteristics of tracheal GCTs.

**Histologic origin**	Mesenchymal (Neurogenic origin from Schwann cell)
**Histologic characteristics**	Large round monomorphic cells with prominent granular eosinophilic cytoplasm
**Immunohistochemical analysis**	Stain positive for S-100 and NSE, negative for smooth muscle, calretinin and inhibin
**Etiology**	Idiopathic (hypothesized role of smoke and estrogen state)
**Differential diagnosis**	Benign tracheal tumors, malignant tracheal tumors, esophageal tumors, thyroid tumors, COPD, bronchial asthma
**Incidence**	Extremely rare, 42 cases reported in literature
**Age predilection**	Third decade of life
**Gender predilection**	Female
**Race predilection**	African and African American
**Clinical features**	Persistent cough, hemoptysis, wheezing, expiratory stridor, obstructive sleep apnea and progressive dyspnea
**CT scan imaging**	Smooth and well-defined margins, elevated contrast enhancement in arterial phase and modest release of contrast in venous phase
**Laryngoscopy and rigid bronchoscopy**	Pedunculated polypoid lesion partially obstructing the lumen
**Treatment**	Surgery (tracheal resection, endoscopic excision, electrosurgery with argon plasma coagulation)
**Prognosis**	Good, primarily affected by airway patency and treatment
**Follow-up**	Annual CT scan and endoscopic examination

These neoplasms are asymptomatic in the vast majority of patients [11]. Clinical features associated with tracheal GCTs include, if present, signs and symptoms such as persistent cough, hemoptysis, wheezing, obstructive sleep apnea and progressive dyspnea unresponsive to beta-2-agonists, glucocorticoids and anticholinergic agents [3]. The initial diagnostic assessment should include a careful patient history and physical examination; many of the tracheal GCTs are diagnosed incidentally on computed tomography (CT) scan or bronchoscopy. Recently, an Italian study focused on the specific pattern of tracheal GCT in multidetector computed tomography scan (MDCT) [16], suggesting that these neoplasms appear to have smooth margins and no sign of invasion at the CT scan imaging without contrast administration. On the other hand, multiphase study shows elevated contrast enhancement in arterial phase and a modest release in venous phase, underlining important differences with other benign tracheal tumors [16]. Endoscopic examinations such as laryngoscopy and bronchoscopy are the diagnostic modalities of choice as they allow for direct visualization for localization, tumor size, luminal status and tissue sampling. Macroscopically, tracheal GCTs appear more frequently as pedunculated polypoid lesion with intact mucosa partially obstructing the tracheal lumen [17]. From a histopathological point of view, GCTs are composed of large round monomorphic cells with eosinophilic and granular cytoplasm [18]; tumor cells usually show a tendency to aggregate in nests without evidence of necrosis or vascular invasion. Immunohistochemically, the expression of S-100 protein and neuron-specific enolase (NSE) is crucial for reaching a definitive diagnosis [19,20]. Tumor cells often express also laminin, HLA-DR, CD56, CD57, CD68 and several myelin proteins [19]; immunohistochemical staining is typically negative for smooth muscle, calretinin and inhibin [20].

Treatments in tracheal GCT include tracheal resection, or endoscopic removal assisted by laser, electrosurgery or argon plasma coagulation [16]. As stated by our search, tracheal resection seems to be particularly effective but the high postoperative mortality rate severely limits its use; on the contrary, endoscopic excision appears to have a not insignificant recurrence rate in absence of perioperative mortality [3]. The need for a radical surgery often includes thyroid lobectomy according to the extension and the involvement of the paratracheal region. Very few data are currently available about the use of laser excision. Given the limited data, a reasonable approach could include endoscopic treatment as initial treatment of choice and tracheal resection in case of recurrent disease or large lesions threatening airway patency. No data are currently available in literature about the use of chemotherapy in tracheal GCTs and no cases of metastasized tracheal GCT have never been reported, further emphasizing the indolent behavior of the disease. As regards the follow-up program, annual CT scans and endoscopic examinations may be recommended although the rarity of the disease and the lack of data about recurrence rate do not permit to define unambiguous recommendations.

## 4 Conclusion

We herein report a case of tracheal GCT in a young woman presenting with dyspnea. Diagnosis of tracheal GCT requires a high index of suspicion given a clinical presentation which often mimics typical signs and symptoms of bronchial asthma or recurrent pneumonia, as in our case. We encourage the need for large case series in order to provide further information concerning tracheal GCTs, taking into account the paucity of data currently available in literature and the extreme rarity of the disease.

## Author contributions

AR: has made substantial contributions to conception of the study, and drafted the manuscript; EDS: has contributed on pathological data and has made substantial contributions to conception of the study; ADR: has helped to draft and revised the manuscript; MN: has been involved in revising the manuscript critically for important intellectual content and have given final approval of the version to be published; MS: has helped to draft and revised the manuscript; AC: has provided the pathological data; DP: has helped to draft and revised the manuscript; RT: has been involved in revising the manuscript critically for important intellectual content, has provided surgical data and have given final approval of the version to be published; Maria AP: have made substantial contributions to conception of the study and has been involved in revising the manuscript critically for important intellectual content and have given final approval of the version to be published.

## Informed consent statement

The patient provided written informed consent.

## Funding

The author(s) received no financial support for the research, authorship, and/or publication of this article.

## Declaration of competing interest

The author(s) declared no potential conflicts of interest with respect to the research, authorship, and/or publication of this article.
